# Accounting for rate-dependent category boundary shifts in speech perception

**DOI:** 10.3758/s13414-016-1206-4

**Published:** 2016-09-14

**Authors:** Hans Rutger Bosker

**Affiliations:** 1 Max Planck Institute for Psycholinguistics, PO Box 310, 6500 AH Nijmegen, The Netherlands; 2 Donders Institute for Brain, Cognition and Behaviour, Radboud University, Nijmegen, The Netherlands

**Keywords:** Speech rate, Rate-dependent perception, Rate normalization, Durational contrast, Neural entrainment

## Abstract

The perception of temporal contrasts in speech is known to be influenced by the speech rate in the surrounding context. This rate-dependent perception is suggested to involve general auditory processes because it is also elicited by nonspeech contexts, such as pure tone sequences. Two general auditory mechanisms have been proposed to underlie rate-dependent perception: durational contrast and neural entrainment. This study compares the predictions of these two accounts of rate-dependent speech perception by means of four experiments, in which participants heard tone sequences followed by Dutch target words ambiguous between /ɑs/ “ash” and /a:s/ “bait”. Tone sequences varied in the duration of tones (short vs. long) and in the presentation rate of the tones (fast vs. slow). Results show that the duration of preceding tones did not influence target perception in any of the experiments, thus challenging durational contrast as explanatory mechanism behind rate-dependent perception. Instead, the presentation rate consistently elicited a category boundary shift, with faster presentation rates inducing more /a:s/ responses, but only if the tone sequence was isochronous. Therefore, this study proposes an alternative, neurobiologically plausible account of rate-dependent perception involving neural entrainment of endogenous oscillations to the rate of a rhythmic stimulus.

Speech can be produced at different rates. The speed at which people speak is known to vary between languages (Pellegrino, Coupé, & Marsico, 2011), between individuals (Quené, 2008), within individuals (Quené, 2013), and even within a single sentence (Miller, Grosjean, & Lomanto, 1984). At the same time, the perception of speech relies heavily on the temporal characteristics of the signal. Many phonological contrasts in the languages of the world involve temporal cues that distinguish the different phonemic categories, such as consonant voicing (voice onset time; VOT), manner of articulation (formant transition duration), gemination, and vowel length (Miller, 1981). As such, variation in the rate at which speech is produced poses a serious challenge to the perceptual system of the listener.

Considering this large-scale variation in speech rate production, listeners are known to interpret speech categories relative to the temporal properties of the surrounding context (henceforth, rate-dependent category boundary shifts). An example of the influence of *proximal context* (i.e., local, typically adjacent, segments) is the finding that the perception of the stop voicing contrast in English (e.g., in /ba/-/pa/; mainly cued by VOT) may be shifted toward /pa/ (longer VOT) when the duration of the following vowel is reduced (Diehl & Walsh, 1989; Kidd, 1989; Miller & Liberman, 1979; Summerfield, 1975). An example of the influence of *distal context* (further removed, typically nonadjacent speech: e.g., the sentence in which a target word is embedded) is the finding that the perception of an /ɑ/-/a:/ continuum in Dutch may be biased toward /a:/ by presenting the target continuum in a fast precursor sentence (Bosker & Reinisch, 2015; Reinisch, 2016a). Similar distal context effects have been found for other temporal contrasts, such as phonological voicing (VOT; Gordon, 1988), manner of articulation (Wade & Holt, 2005), lexical stress (Reinisch, Jesse, & McQueen, 2011a), and word segmentation (Reinisch, Jesse, & McQueen, 2011b).

The literature seems to suggest that rate-dependent category boundary shifts are to be explained by a general auditory mechanism. This claim is supported by evidence that rate-dependent perception is found in young infants (Eimas & Miller, 1980) and in nonhuman species (Welch, Sawusch, & Dent, 2009). Moreover, rate-dependent effects occur very early in perceptual processing (Reinisch, 2016b; Reinisch & Sjerps, 2013) and are not modulated by cognitive load (Bosker, Reinisch, & Sjerps, 2016). Finally, rate-dependent effects are sensitive to the rate of speech produced by nontarget talkers (Bosker, 2016; Newman & Sawusch, 2009) and even to the rate of nonspeech precursors (e.g., fast vs. slow pure tone sequences; Gordon, 1988; Wade & Holt, 2005; but see Pitt, Szostak, & Dilley, 2016).

Two possible general auditory mechanisms have been proposed to account for rate-dependent category boundary shifts. The first account is the principle of *durational contrast*, which was introduced by Diehl and Walsh (1989). They stipulated that the “perceived length of a given acoustic segment is affected contrastively by the duration of adjacent segments” (p. 2154). That is, a target phonetic duration will be perceived as longer in the context of shorter segments than in the context of longer segments.

The principle of durational contrast was originally formulated to explain proximal context effects (of adjacent segments), but has also been suggested to explain distal context effects (of sentential rate). For instance, Wade and Holt (2005) presented participants with a /ba/-/wa/ continuum (varying formant transition duration) preceded by two particular tone sequences: a fast tone sequence (short tones presented at a fast rate) or a slow tone sequence (long tones presented at a slow rate). Across two experiments (with various amplitude manipulations), the authors observed that the fast-tone sequence biased the perception of target words toward /wa/. The authors took this result as evidence for durational contrast, with the duration of the tones exerting a contrastive influence on the perception of the following ambiguous initial consonant.

Another general auditory account of rate-dependent perception involves *neural entrainment* to the syllabic rhythm of speech. The neurocognitive literature indicates that the brain tracks incoming speech by phase-locking intrinsic oscillators to the syllabic rhythm of the speech signal (Giraud & Poeppel, 2012). The (approximately syllabic) amplitude fluctuations present in speech are thought to elicit a phase reset of cortical oscillations, which thereafter track the speech envelope (Doelling, Arnal, Ghitza, & Poeppel, 2014; Luo & Poeppel, 2007; Peelle, Gross, & Davis, 2013). Thus, neuronal excitability is temporally aligned with the temporal structure of the acoustic input, serving as a parsing mechanism for the initial neural coding of the speech signal (Arnal, Giraud, & Poeppel, 2015; Gross et al., 2013).

Recent studies (e.g., Dilley & Pitt, 2010; Peelle & Davis, 2012; Pitt et al., 2016) have alluded to neural entrainment as a potential explanatory mechanism behind rate-dependent perception, particularly distal context effects of sentential rate (although empirical evidence is currently lacking). For instance, Peelle and Davis (2012) have suggested that there is a consistent phase relationship between the onset of phonetic segments and ongoing (entrained) cortical oscillations, guiding speech perception. To exemplify, the segmental onset for /p/ may hypothetically occur consistently in the low-excitability phase of entrained oscillations, whereas the segmental onset for /b/ may consistently occur in the high-excitability phase (cf. Figure 6 in Peelle & Davis, 2012). Neural entrainment to a fast sentential context induces shifts in the relationship between segmental onsets and oscillatory phase. Thus, the segmental onset of an ambiguous bilabial stop, following a fast precursor, may fall in a more low-excitability phase of entrained oscillations (vs. in a more high-excitability phase following a slow precursor), biasing perception toward /p/ after fast speech.

This study aims to contribute to our understanding of rate-dependent category boundary shifts in speech perception by comparing predictions from the two general auditory accounts introduced above: durational contrast and neural entrainment. This comparison primarily concerns distal context effects of sentential. Crucially, the two accounts differ with respect to the cue in the acoustic context that is thought to elicit these distal context effects: the duration of surrounding acoustic units (i.e., in ms) or their presentation rate (i.e., number of units per second, in Hz). Of course, in natural speech, syllabic durations and speech rate covary: faster speech typically contains shorter syllables. Nevertheless, in a lab experiment, duration and rate can easily be separated by manipulating intervening silent intervals, which allows for discrimination of predictions from the two accounts of rate-dependent perception.

Specifically, this study adopted the experimental design from Wade and Holt (2005): Participants were presented with pure tone sequences (precursors) followed by a vowel continuum between Dutch /ɑ/ and /a:/ (targets). In Experiments 1–3 (using various sample sizes and various vowel continua), four precursor conditions were used—namely, precursors containing tones with either short or long durations, presented at either a fast or a slow presentation rate. Using this full factorial design, the independent (and potentially combined) contributions of tone duration and presentation rate may be disentangled.

If we follow the principle of durational contrast, then modulating the duration of the tones in the tone precursor should elicit rate-dependent category boundary shifts, independent of the presentation rate of the tones (specifically, shorter tones would bias target perception toward /a:/). In contrast, oscillation-based models of speech perception state that intrinsic oscillators phase-lock specifically to the rate of a particular acoustic precursor (Doelling et al., 2014; Ghitza, 2014). Therefore, if we follow proposals about neural entrainment, then rate-dependent perception should be elicited by modulating the presentation rate of the tones in the precursor, independent of the duration of those tones (specifically, faster rates would bias target perception toward /a:/). Note, however, that the two accounts are not mutually exclusive; in fact, they may operate in tandem, with both duration modulations and rate modulations affecting speech perception.

Finally, proposals about neural entrainment maintain a central role for the rhythmic nature of speech in rate-dependent perception. If Experiments 1–3 find that modulating the precursors’ presentation rate elicits rate-dependent perception, then removing the regular timing of a tone sequence may eliminate the effect of different presentation rates. If, however, durational contrast induces rate-dependent perception, then the (regular or irregular) timing of a tone sequence should not influence perception. Experiment 4 compared the effect of isochronous tone precursors (as used in Experiments 1–3) to the effect that anisochronous tone precursors (i.e., with jittered interonset intervals) might have on target perception.

## Experiment 1

### Method

The experimental design of this study resembles the design introduced in Wade and Holt (2005). However, here, the Dutch vowel contrast between /ɑ/ and /a:/ was investigated instead of the English /ba/-/wa/ contrast in Wade and Holt (2005).

#### Participants

Similar sample sizes as those used in Wade and Holt (2005) were adopted. Native Dutch participants (*N* = 14, two males, 12 females, *M*_age_ = 33 years) with normal hearing were recruited from the Max Planck Institute (MPI) participant pool, with informed consent as approved by the Ethics Committee of the Social Sciences Department of Radboud University (Project Code: ECSW2014-1003-196).

#### Design and materials

**Fig. 1:**
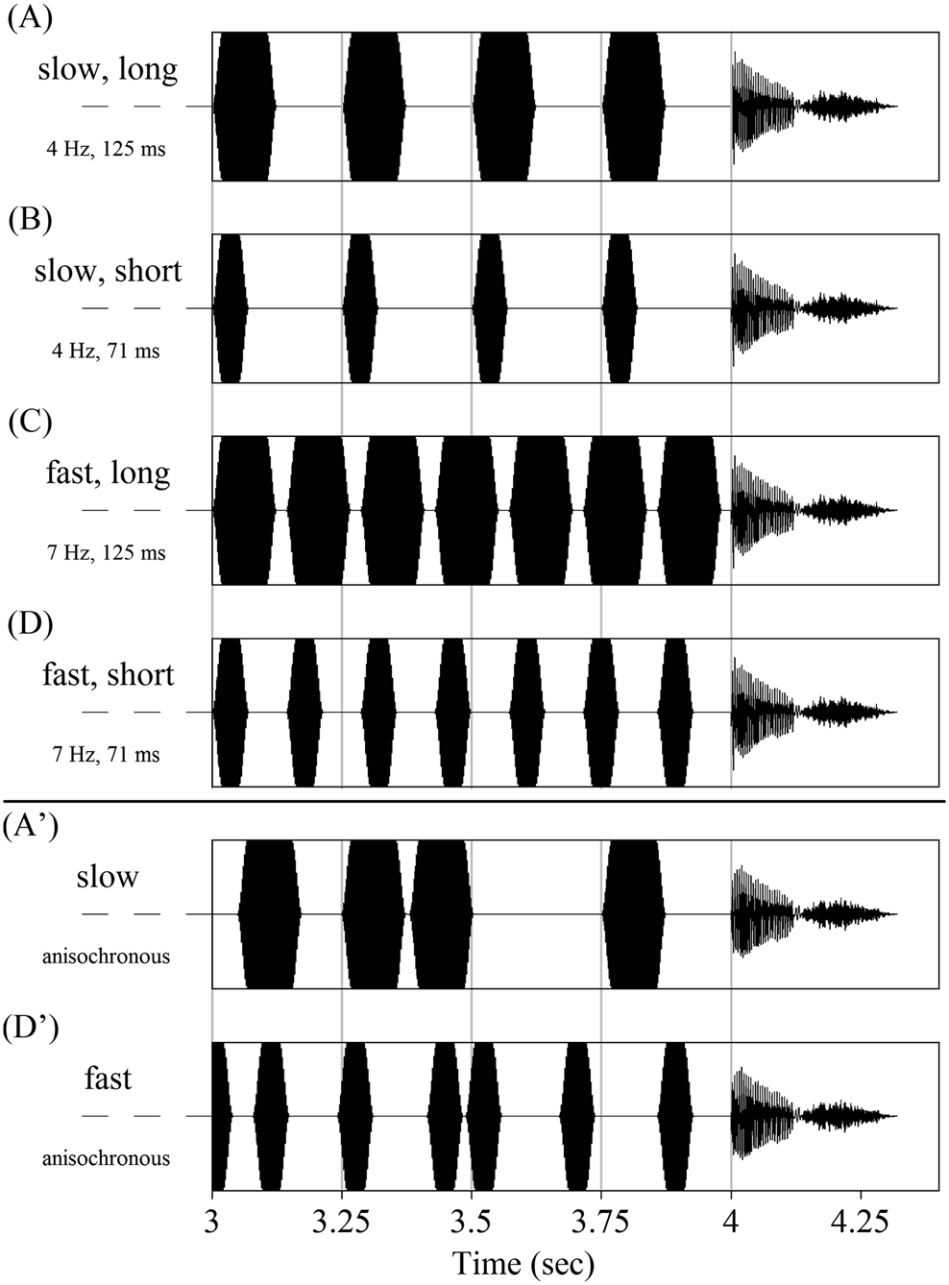
Examples of the precursor conditions used the different experiments. The top panel (Conditions A, B, C, and D) shows the isochronous precursor conditions used in Experiments 1–3; the bottom panel shows the anisochronous precursor conditions used in Experiment 4. Each plot shows the final second of a tone sequence (total duration = 4 s), followed by a target word, with the precursor condition given to the left

The stimuli in the experiment consisted of tone precursors followed by target words (see Fig. 1). Four different precursors, each with a total duration of 4 seconds, were created in Praat (Boersma & Weenink, 2012) by crossing two different tone durations (71 vs. 125 ms) with two presentation rates (4 vs. 7 Hz):

A. SLOW, LONG: tones of 125 ms presented at a rate of 4 Hz
B. SLOW, SHORT: tones of 71 ms presented at a rate of 4 Hz
C. FAST, LONG: tones of 125 ms presented at a rate of 7 Hz
D. FAST, SHORT: tones of 71 ms presented at a rate of 7 Hz

The presentation rates were selected to fall within the range of typical speech rates. The tone durations (including a 20-ms rise-and-decay time) were selected to fall within the range of typical durations of the vowels /ɑ/ and /a:/ in Dutch, and were derived from the selected rates using the formula: 1/(2 × rate). The fundamental frequency of all pure tones was fixed at 440 Hz, thus avoiding spectral masking of the target vowels’ F0, F1, and F2. Because the phase relationship between target word onset and an acoustic periodic precursor may influence perception (ten Oever & Sack, 2015), target word onset was kept at a constant phase (0 degrees) across the different precursors.

For the target words, a female native speaker of Dutch was recorded, producing the Dutch minimal word pair *as* /ɑs/ “ash” and *aas* /a:s/ “bait.” From these recordings, one long vowel /a:/ was selected for manipulation. Because the Dutch /ɑ/-/a:/ contrast is cued by both spectral and temporal characteristics, a two-dimensional continuum was created from this one vowel token, comprising five duration values and five F2 values, all falling within the speaker’s natural range. Spectral manipulations were based on Burg’s LPC method (implemented in Praat), with the source and filter models estimated automatically from the selected vowel. The formant values in the filter models were inspected and adjusted to result in a constant F1 value (810 Hz, ambiguous between /ɑ/ and /a:/) and one of five desired F2 values (1350–1550 Hz in steps of 50 Hz). Then, the source and filter models were recombined and the new vowels were adjusted to have the same overall amplitude as the original vowel. Based on these spectrally manipulated vowels, duration continua (120–160 ms in steps of 10 ms) were created using PSOLA. Finally, the vowel tokens were combined with one single /s/ token (set to a constant duration of 200 ms) to form 25 manipulated target words.

These target words were presented *in isolation* (i.e., without any precursor) to 11 native Dutch listeners in a categorization pretest (two-alternative forced choice; none of these participants took part in any of the other experiments). Listeners indicated whether they heard the word *as* or *aas*. Based on this pretest, four vowel tokens with different F2 values but identical duration (120 ms) were selected, each sampling a different point from the categorization curve: Token 1, F2 = 1400 Hz, 14 % /a:/-categorization; Token 2, F2 = 1450 Hz, 25 % /a:/-categorization; Token 3, F2 = 1500 Hz, 45 % /a:/-categorization; and Token 4, F2 = 1550 Hz, 77 % /a:/-categorization. Target words with only these four vowel tokens were used in the following experiment. Finally, the target words were combined with the four different precursors. Each stimulus was presented 10 times per session (total number of trials: 160).

#### Procedure

Stimulus presentation was controlled by Presentation software (Version 16.5; Neurobehavioral Systems, Albany, CA, USA). Stimuli were presented to half of the participants in a fixed random order, with the reversed order presented to the other half, and participants were allowed to take a short break halfway through the experiment.

Each trial started with a fixation cross appearing in the middle of the screen. After 330 ms, the auditory stimulus was presented. At target offset, the fixation cross was replaced by the two response options *as* and *aas* on the left and right side of the screen (position counterbalanced across participants), and participants were instructed to indicate by button press which target word they had heard (“1” for the left word and “0” for the right word). If participants did not respond within 4 seconds, a missing response was recorded, and the next trial was presented.

### Results

**Fig. 2:**
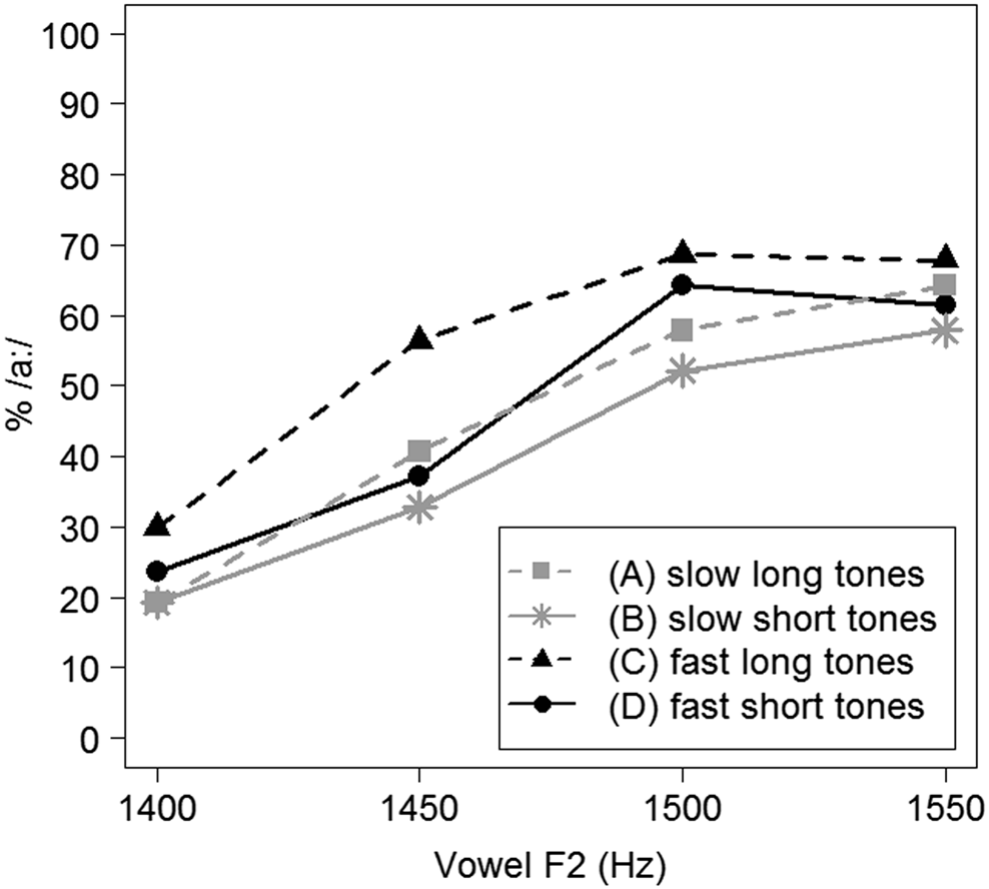
Average categorization data (in % /a:/ responses) for Experiment 1, split by four different precursor conditions

Categorization data, calculated as the percentage of /a:/ responses (% /a:/), of Experiment 1 are represented in Fig. 2.

A generalized linear mixed model with a logistic linking function as implemented in the lme4 library in R (Bates, Maechler, & Bolker, 2015) tested the binomial responses (/a:/ = 1; /ɑ/ = 0) for fixed effects of Vowel F2 (continuous predictor, scaled around the mean), Tone Duration (categorical predictor, with the short duration of 71 ms mapped onto the intercept), Presentation Rate (categorical predictor, with the slow rate of 4 Hz mapped onto the intercept), and all their interactions, with random effects of Participants. By-participant random slopes for all fixed effects and all interactions were included in the model.

This model revealed significant effects of vowel F2 (the higher the vowel’s F2, the higher the percentage of /a:/ responses; *β* = 1.013, *z* = 4.067, *p <* .001) and of presentation rate (a higher percentage of /a:/ responses for trials with precursors with a presentation rate of 7 Hz; *β* = 0.490, *z* = 2.440, *p =* .015). No effect of tone duration could be established (*p >* 0.2), nor was any interaction between any of the predictors observed.

### Discussion

In Experiment 1, participants listened to target words ambiguous between /ɑs/ and /a:s/, preceded by tone sequences with either short or long tones, presented at either a fast or a slow presentation rate. The use of a full factorial design, crossing the factors Tone Duration and Presentation Rate, allowed for distinguishing whether the precursors’ tone duration and/or the precursors’ presentation rate induces a shift in the phonetic category boundary between /ɑ/ and /a:/.

The results of Experiment 1 are inconsistent with a durational contrast account of rate-dependent perception because no effects of varying tone durations were found. Instead, faster presentation rates biased listeners’ categorization responses towards /a:/, corroborating claims from proposals about neural entrainment.

Nevertheless, even though no significant effect of tone duration could be found, there would seem to be an apparent difference between precursors with short and long tones in Fig. 2. Note, however, that this dissimilarity is in the opposite direction from what the durational contrast account would hypothesize. Moreover, the statistically significant difference between precursors with a fast versus a slow presentation rate would seem to be rather variable across the vowel continuum.

Motivated by this apparent variability in the data of Experiment 1 and the present drive for replicability in psychological science (Open Science Collaboration, 2015), a second experiment was designed. Experiment 2 adopts the procedure of Experiment 1, with larger sample sizes to increase statistical power, thus increasing the generalizability of results and aiding the interpretation of potential variability in the data.

## Experiment 2

### Method

#### Participants

A sample of 18 native Dutch participants (six males, 12 females, *M*_age_ = 38 years) with normal hearing was recruited from the MPI participant pool, with informed consent as approved by the Ethics Committee of the Social Sciences Department of Radboud University (Project Code: ECSW2014-1003-196).

#### Design

The experimental design of Experiment 2 was identical to that of Experiment 1, except that in Experiment 2 participants were presented with twice as many trials (i.e., 20 repetitions of each unique stimulus; 320 trials in total).

### Results

**Fig. 3:**
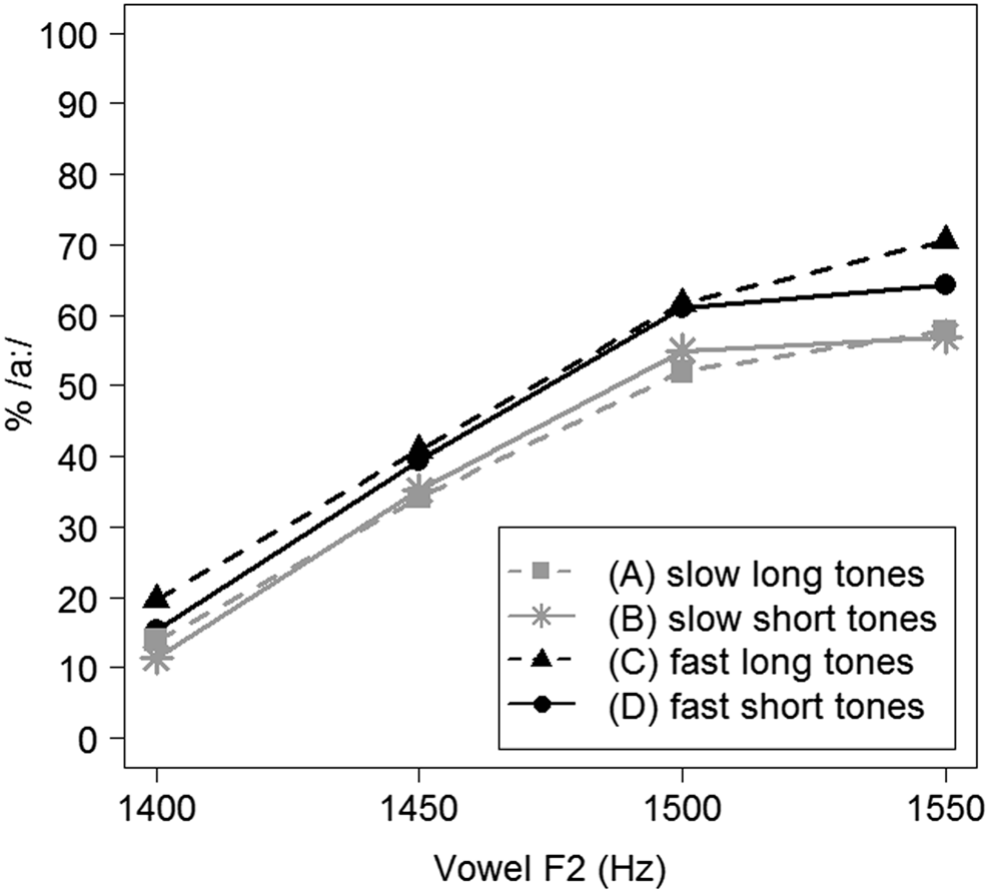
Average categorization data (in % /a:/ responses) for Experiment 2, split by four different precursor conditions

Categorization data, calculated as the percentage of /a:/ responses (% /a:/), of Experiment 2 are represented in Fig. 3.

Another generalized linear mixed model, with a logistic linking function and an identical structure as the previously described model, tested the binomial responses (/a:/ = 1; /ɑ/ = 0) from Experiment 2. This model revealed significant effects of Vowel F2 (the higher the vowel’s F2, the higher the percentage of /a:/ responses; *β* = 0.841, *z* = 5.626, *p <* .001) and of Presentation Rate (a higher percentage of /a:/ responses for trials with precursors with a presentation rate of 7 Hz; *β* = 0.475, *z* = 2.773, *p =* .006). No effect of Tone Duration could be established (*p >* .9). One interaction, between Vowel F2 and Presentation Rate, was found (*β* = 0.289, *z* = 2.034, *p =* .042), indicating a larger effect of the precursor’s presentation rate for target tokens with a higher vowel F2. This interaction is likely due to a floor effect in vowel tokens with lower F2 values because context effects in speech perception are typically most pronounced when local phonetic cues are ambiguous.

### Discussion

The results from Experiment 2 mirror the results from Experiment 1: no effect of tone duration was observed, but, instead, faster presentation rates shifted the category boundary between the two target vowels. The absence of effects of tone duration in both experiments challenges durational contrast as explanatory mechanism behind rate-dependent category boundary shifts. However, note that the target vowel continuum in both experiments concerned a spectral continuum, varying F2 while keeping duration constant. Because target vowel duration was uninformative to the categorization task in both experiments, this may have led listeners to give little perceptual weight to the duration of preceding tones.

To investigate this issue further, a third experiment was designed. Because the Dutch /ɑ/- /a:/ contrast is cued by both spectral and temporal characteristics, Experiment 3 was identical to Experiment 2, except that, this time, a two-dimensional (spectral and temporal) target vowel continuum was used.

## Experiment 3

### Method

#### Participants

A sample of 25 native Dutch participants with normal hearing was recruited from the MPI participant pool, with informed consent as approved by the Ethics Committee of the Social Sciences Department of Radboud University (Project Code: ECSW2014-1003-196). Data from four participants were excluded for reasons of technical failures or noncompliance, leaving data from 21 participants for analysis (six males, 15 females, *M*_age_ = 24 years).

#### Design and materials

The experimental design of Experiment 3 was identical to that of Experiment 2, except that in Experiment 3 participants were presented with a two-dimensional target vowel continuum, varying both vowel duration and vowel F2 (cf. pretest described in Experiment 1). Vowel duration varied from 120 ms to 150 ms in four steps of 10 ms and F2 varied from 1400 Hz to 1550 Hz in four steps of 50 Hz. The same four precursors were used as in the previous experiments, resulting in a total number of 64 unique stimuli (4 precursors × 16 target vowels). To keep the total number of trials identical to that in Experiment 2, each unique stimulus was presented five times to each participant.

#### Procedure

The procedure in Experiment 3 was identical to the previous experiments: participants were presented with tone sequences, followed by target words, and indicated by button press which target word they had heard.

### Results

**Fig. 4:**
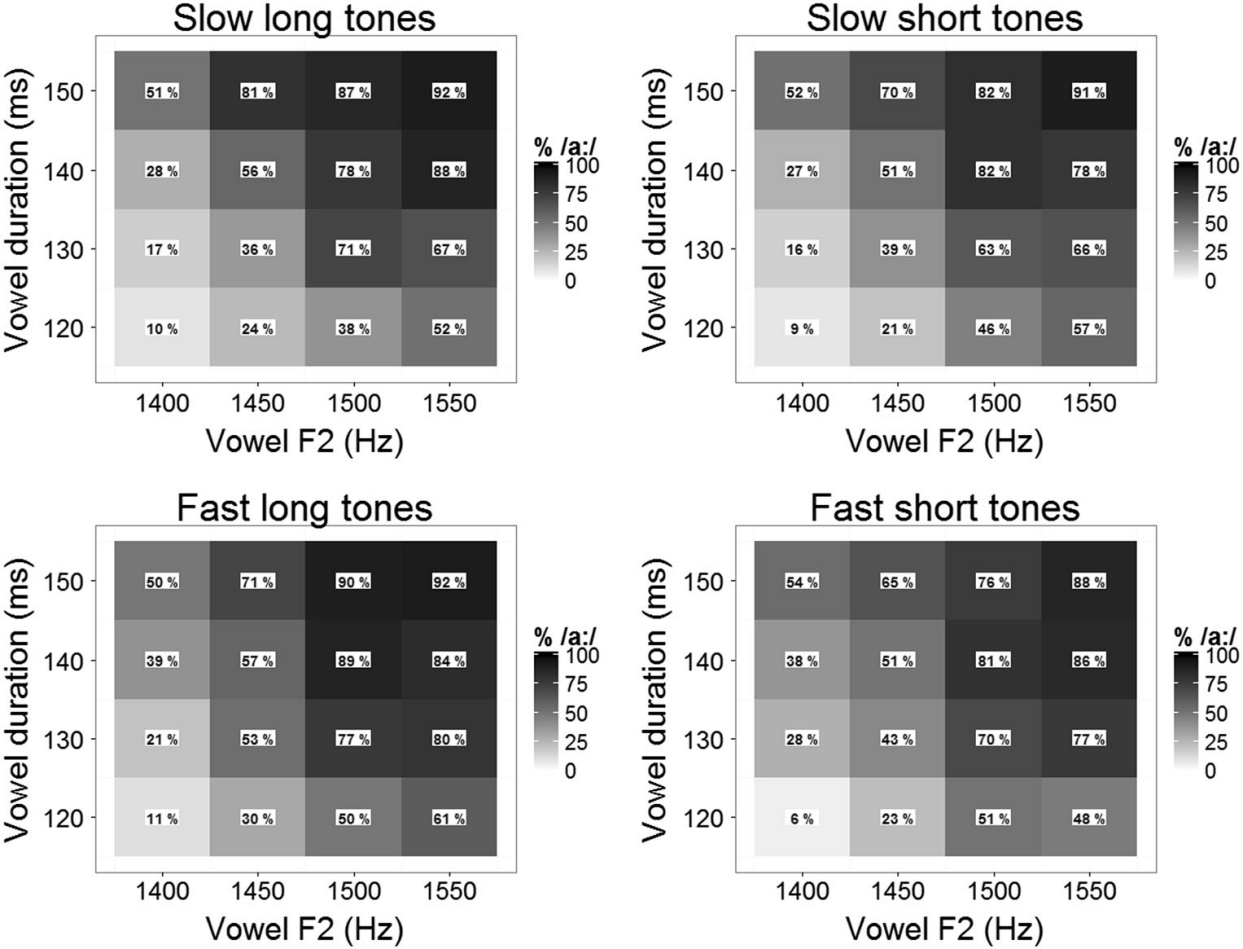
Results of Experiment 3. Each panel displays % /a:/ responses to the two-dimensional vowel continuum (F2 on *x*-axis, duration on *y*-axis) for one of four different precursor conditions (slow long, slow short, fast long, fast short). For each combination of duration and F2 value (each tile), the tint reflects the % /a:/ responses indicated in the tile (the darker the tint, the higher the % /a:/ responses)

Figure 4 shows the categorization data (% /a:/) of Experiment 3 for each step on the two-dimensional vowel continuum and each precursor condition. The percentage of /a:/ responses can be seen to increase (darker tint of the tiles) as vowel duration and vowel F2 increase. Also, the bottom two panels, showing the data for the two fast precursor conditions (presentation rate of 7 Hz) are slightly darker than the top two panels, showing the data for the two slow precursor conditions (presentation rate of 4 Hz). Categorization data for the different precursor conditions, collapsing across the vowel continuum: SLOW, LONG: 55 %; SLOW, SHORT: 53 %; FAST, LONG: 59 %; FAST, SHORT: 55 %.

A generalized linear mixed model with a logistic linking function tested the binomial responses (/a:/ = 1; /ɑ/ = 0) from Experiment 3 for fixed effects of Vowel F2 (continuous predictor, scaled around the mean), Vowel Duration (continuous predictor, scaled around the mean), Tone Duration (categorical predictor, with the short duration of 71 ms mapped onto the intercept), Presentation Rate (categorical predictor, with the slow rate of 4 Hz mapped onto the intercept), and all their interactions, with random effects of Participants. By-participant random slopes for all fixed effects and all interactions were included in the model.

This model revealed significant effects of Vowel F2 (the higher the vowel’s F2, the higher the percentage of /a:/ responses; *β* = 1.266, *z* = 7.160, *p <* .001) and Vowel Duration (the longer the vowel’s duration, the higher the percentage of /a:/ responses; *β* = 1.172, *z* = 10.499, *p <* .001), showing that participants used both temporal and spectral cues to distinguish between the two vowel categories. Also, an effect of Presentation Rate was observed (a higher percentage of /a:/ responses for trials with precursors with a presentation rate of 7 Hz; *β* = 0.424, *z* = 2.596, *p =* .009). No effect of Tone Duration could be established (*p >* .2).

### Discussion

The results from Experiment 3 mirror the results from the other experiments: faster presentation rates shifted the category boundary between the two target vowels. Even though Experiment 3 used a two-dimensional (spectral and temporal) target vowel continuum (i.e., with duration being an informative cue to vowel categorization), no effect of tone duration could be established.

None of the experiments so far found an effect of the duration of tones in the precursors. In contrast, all experiments found evidence that the faster presentation rate biased perception toward /a:/, corroborating claims from proposals about neural entrainment. A final test of the influence of precursors’ presentation rate on target perception involves the comparison between isochronous (regular interonset intervals) and anisochronous (*ir*regular interonset intervals) tone sequences. Removing the strictly regular timing of the tone sequences would be predicted to eliminate the presentation rate effect previously observed. Therefore, in Experiment 4, participants were presented with isochronous and anisochronous tone sequences.

## Experiment 4

### Method

The experimental design of Experiment 4 resembled that of the previous experiments. Once more, target words ambiguous between /ɑs/ *ash* and /a:s/ *bait* were presented after tone sequences. However, this time the effect of isochronous precursors (with short or long tones occurring at regular intervals) was compared to the effect that anisochronous precursors (with short or long tones occurring at irregular intervals) would have on target perception.

#### Participants

Native Dutch participants (*N* = 26) with normal hearing were recruited from the MPI participant pool, with informed consent as approved by the Ethics Committee of the Social Sciences Department of Radboud University (Project Code: ECSW2014-1003-196). Data from two participants were lost due to technical errors, leaving data from 24 participants available for analysis (seven males, 17 females, *M*_ag*e*_ = 23 years).

#### Design and materials

Similar to the previous experiments, the stimuli in Experiment 4 consisted of tone precursors followed by target words. However, this time the crucial comparison involved the differential contribution of isochronous versus anisochronous tone precursors to target word perception. Therefore, Condition A (slow, long) and Condition D (fast, short) were adopted from the previous experiments (Conditions B and C did *not* form part of Experiment 4). Furthermore, two new precursor conditions were created that were identical to Conditions A and D, except that the interonset interval (IOI) between individual tones was randomized (Conditions A’ and D’, respectively; see Fig. 1). This manipulation removed the isochronous character of the precursors without changing the precursors’ total duration, the duration of individual tones, or the total number of tones. Each trial in Condition A’ or D’ had a unique random IOI pattern (A’: mean IOI = 125 ms, *SD* = 71 ms; D’: mean IOI = 71 ms, *SD* = 39 ms). To avoid energetic masking and to aid the comparison between isochronous and anisochronous precursor conditions, the temporal position of the final tone in the sequence was left unchanged. That is, in both the isochronous (A and D) and the corresponding anisochronous conditions (A’ and D’), the silent interval preceding the target word was kept constant.

Finally, the same spectral (unidimensional) target continuum as used in Experiments 1 and 2 was adopted. Each target word appeared in all four precursor conditions. Similar to Experiment 2, each unique stimulus was presented 20 times to each participant.

#### Procedure

The procedure in Experiment 4 was identical to the previous experiments: Participants were presented with tone sequences, followed by target words, and indicated by button press which target word they had heard.

### Results

**Fig. 5:**
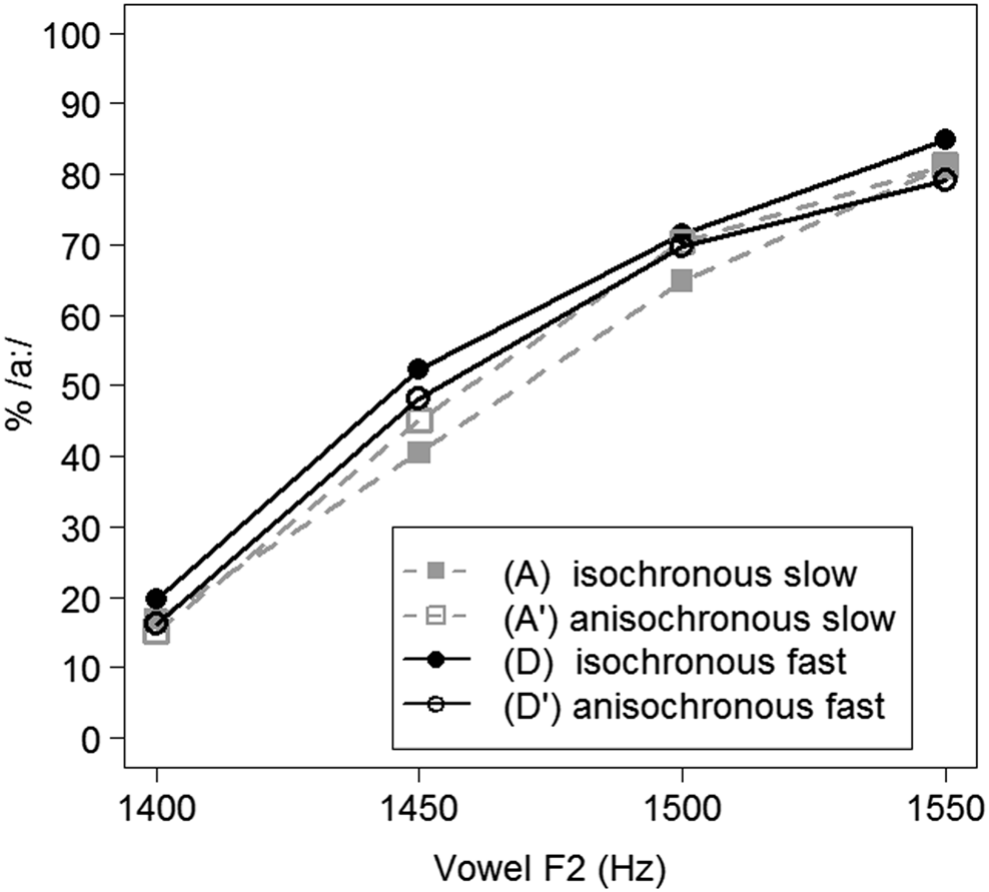
Average categorization data (in % /a:/ responses) for Experiment 4, split by four different precursor conditions

Categorization data, calculated as the percentage of /a:/ responses (% /a:/), of Experiment 4 are represented in Fig. 5.

A generalized linear mixed model with a logistic linking function tested the binomial responses from Experiment 4 for fixed effects of Vowel F2 (continuous predictor, scaled around the mean), Tone Duration (categorical predictor, with the short duration of 71 ms mapped onto the intercept), Isochrony (categorical predictor, with “anisochronous” mapped onto the intercept), and all their interactions. Random effects of Participants and by-participant random slopes for all fixed effects and interactions were included in the model.

This model revealed significant effects of Vowel F2 (the higher the vowel’s F2, the higher the percentage of /a:/ responses; *β* = 1.427, *z* = 7.365, *p <* .001) and of Isochrony (isochronous tones, Condition D, received a higher percentage of /a:/ responses relative to the anisochronous version, Condition D’; *β* = 0.304, *z* = 2.635, *p <* .001), but no effect of Tone Duration (*p >* .9). One interaction, between Isochrony and Tone Duration, was found (*β* = -0.482, *z* = -2.772, *p =* .006), indicating a difference between the isochronous Conditions A and D (faster presentation rates biasing perception towards more /a:/ responses) which was absent in the anisochronous conditions.

### Discussion

In Experiment 4, participants were presented with tones presented at fast and slow presentation rates, at either regular (isochronous precursors) or irregular intervals (anisochronous precursors). Target word perception was observed to be sensitive to the precursor’s presentation rate, with fast presentation rates biasing perception towards more /a:/ responses, replicating the findings from the previous two experiments. However, the effect of the precursor’s presentation rate was eliminated when the regular timing of the tones in the precursors was removed (i.e., in the anisochronous precursor conditions).

The principle of durational contrast cannot explain these findings because it only makes reference to the duration of surrounding segments, independent of the isochronous character of precursors. Instead, it would appear that listeners’ ability to track the temporal regularities in the acoustic signal influenced perception.

## General Discussion

These experiments were designed to extend our understanding of the explanatory mechanism behind rate-dependent category boundary shifts in speech perception. Two general auditory accounts of rate-dependent perception were distinguished: durational contrast and neural entrainment. These two accounts crucially differ with respect to the cue in the acoustic context that is thought to elicit rate-dependent perception. In durational contrast, the *duration* of surrounding acoustic units plays a central role. In contrast, proposals about neural entrainment stress the role of intrinsic oscillations that phase lock to the *rate* of spoken input. By presenting participants with tone precursors with different tone durations and different presentation rates, the predictions of the two accounts may be compared.

In the first three experiments (using various sample sizes and various vowel continua), a consistent effect of the precursors’ presentation rate was found, with faster presentation rates biasing target perception toward /a:/. In contrast, no effect of tone duration was found in any of the experiments. This finding challenges durational contrast as explanatory mechanism behind rate-dependent category boundary shifts. Note that the two accounts of rate-dependent perception are not mutually exclusive: an effect of presentation rate does not preclude an additional effect of tone duration. Nevertheless, no evidence for contrastive effects of surrounding durations was found.

This observation may first of all be interpreted to call for a reinterpretation of the results reported in Wade and Holt (2005). In that study, participants were also presented with fast and slow tone precursors and, similar to these results, fast precursors were observed to bias subsequent target perception toward longer segments. This result was taken as evidence for durational contrast, with the duration of the tones exerting a contrastive influence on target perception. However, in the fast and slow tone sequences used by Wade and Holt (2005), tone duration and presentation rate covaried (i.e., the short tone sequence had a high presentation rate and the long tone sequence had a slow presentation rate). Therefore, as the authors also acknowledge, the contribution of tone duration could not be disentangled from the contribution of the tones’ presentation rate. Given the present findings, it would seem that the sequences’ presentation rate, and not durational contrast, may be held responsible for the rate-dependent effects observed in Wade and Holt (2005).

Because the current experiments were built upon the method of Wade and Holt (2005), both Wade and Holt (2005) and this study used nonspeech precursors (pure tone sequences). Although the use of nonspeech constrains comparison with spontaneous conversation, it emphasizes the general auditory nature of rate-dependent category boundary shifts. In that respect, this study is in line with Wade and Holt (2005): Rate-dependent category boundary shifts are elicited by both speech and nonspeech contexts.

However, it seems that not all rate effects in speech perception are domain general. Consider, for instance, the lexical rate effect (LRE), observed when heavily coarticulated function words (such as “or” in a phrase like “leisure or time”) seem to disappear when presented in a slow context sentence (Dilley & Pitt, 2010). Recently, Pitt et al. (2016) have demonstrated that the LRE arises in intelligible contexts (e.g., in clear speech; primed, and therefore intelligible, low-pass filtered speech; primed sinewave speech) but *not* in unintelligible contexts (e.g., pure tones; unprimed low-pass filtered speech; unprimed sinewave speech). What exactly differentiates (domain-general) rate-dependent category boundary shifts from the (domain-specific) LRE is unclear. It may be speculated that the different rate effects operate at different processing levels, with rate effects on lower (segmental) levels being domain general (i.e., category boundary shifts), while influences at higher (lexical) levels only occur in linguistically intelligible contexts (i.e., the LRE). This remains speculative, however, and future studies will have to clarify how the mechanisms underlying the LRE and rate-dependent category boundary shifts relate to each other.

In Experiment 4, participants were presented with fast and slow presentation rates in isochronous (with regular IOIs) and anisochronous tone sequences (with irregular IOIs). Presentation rate modulations only affected target perception in isochronous precursors, not in anisochronous precursors. This finding emphasizes the central role of temporal regularities in rate-dependent speech perception, in line with proposals in the neurocognitive literature about neural entrainment.

This neurocognitive literature suggests that the brain aligns the phase of endogenous neural oscillators to (quasi-)periodicities present in sensory signals, such as periodic light flashes (Busch, Dubois, & VanRullen, 2009) or rhythmic tone sequences (Stefanics et al., 2010). Even though naturally produced speech is not a purely periodic signal, neural oscillations within the *theta* range (3–8 Hz; Giraud & Poeppel, 2012) can track the slow (approximately syllabic) amplitude modulations in speech (Ding, Melloni, Zhang, Tian, & Poeppel, 2016; Luo & Poeppel, 2007). Thus, neural entrainment shapes speech perception by imposing particular frequencies and phases of high and low temporal sampling onto the stimulus-driven neural spike train (Lakatos et al., 2005). This is corroborated by findings of increased sensitivity (faster responses, higher accuracy) for stimuli that occur in high excitability phases of the entrained rhythm (Busch et al., 2009; Henry & Obleser, 2012; Zoefel & VanRullen, 2015). In this fashion, neural entrainment acts to sample the input signal at the appropriate temporal granularity: denser sampling of faster signals and sparser sampling of slower signals (Giraud & Poeppel, 2012).

These proposals may be argued to account for the findings of this study. The isochronous tone sequences, used in the current experiments, may have induced entrainment of neural oscillations, as commonly observed in electrophysiological studies using isochronous tone sequences (Gomez-Ramirez et al., 2011; Lakatos et al., 2005; Stefanics et al., 2010). Thus, participants’ neural oscillations were entrained at a higher frequency by isochronous tone sequences with a fast presentation rate than by isochronous tone sequences with a slow presentation rate. This differential neural entrainment may be presumed to have induced distinct sampling regimes, with denser sampling following from tone sequences with faster presentation rates. Consequently, a higher sampling rate would lead to an overestimation of the target duration, biasing perception toward the long vowel /a:/ (as observed in Experiments 1–3). Similarly, lower sampling rates, induced by slow tone sequences, would underestimate the target’s duration, biasing perception towards /ɑ/. Moreover, removing the temporal regularities from an acoustic signal reduces the fidelity of auditory entrainment to the signal (Kayser, Ince, Gross, & Kayser, 2015). As a consequence, anisochronous signals do not induce consistent neural sampling regimes, eliminating the biasing effect of slow and fast presentation rates (as observed in Experiment 4).

This account of the rate-dependent category boundary shifts, with reference to over- and undersampling of target speech sounds, contrasts with the account proposed by Peelle and Davis (2012), which is also grounded in oscillation-based models of speech perception. In Peelle and Davis’ proposal, shifts in the phase relationship between segmental onset and cortical oscillators are responsible for rate-dependent perception. However, for these experiments, the relationship between target onset and the phase of the presumed entrained neural rhythms was constant across all conditions (consistent phase of 0 degrees). Therefore, it is difficult to see how the proposal by Peelle and Davis (2012) can account for these findings. Nevertheless, the question whether varying the phase of target sound presentation elicits additional perceptual changes is an intriguing topic for further investigation (cf. ten Oever & Sack, 2015).

Now, how do these findings, obtained with nonspeech precursors with strictly isochronous pure tones, scale up to speech perception in natural conversation? Clearly, spontaneously produced speech is not as periodic as highly controlled isochronous tone sequences. Rather, the rate at which natural speech is produced varies considerably (Miller et al., 1984). Nevertheless, even with all its spontaneous temporal variation, speech is a pseudorhythmic signal with slow amplitude modulations in the *theta* range (3–8 Hz; Giraud & Poeppel, 2012). This pseudorhythmic nature of speech is found across typologically diverse languages (Greenberg & Arai, 2004; Ohala, 1975; Pellegrino et al., 2011) and is critical for accurate speech perception: if the *theta* rhythm is destroyed or filtered out, intelligibility drops considerably (Ghitza, 2012, 2014; Ghitza & Greenberg, 2009). Therefore, even though spontaneous speech is not as rhythmic as the perfectly isochronous precursors used here, it is argued that the pseudorhythmic nature of speech is sufficient to trigger the oscillatory mechanism (neural entrainment) suggested to be responsible for rate-dependent speech perception.

These experiments could not find any evidence for contrastive effects of surrounding durations, suggesting that contrastive perception of duration cues does not contribute to the *distal* rate effect investigated here. Durational contrast was originally formulated to account for *proximal* context effects, such as adjacent vowel durations influencing the perception of surrounding consonants (Diehl & Walsh, 1989; Kidd, 1989; Miller & Liberman, 1979; Newman & Sawusch, 1996; Sawusch & Newman, 2000). However, the role of durational contrast in proximal context effects has recently been challenged by evidence for cue-integration frameworks (Toscano & McMurray, 2012, 2015). In such frameworks, the duration of the vowel following a stop onset is seen as an independent cue to the stop’s voicing specification (rather than contrastively influencing the perception of the stop’s VOT; Toscano & McMurray, 2012). Clearly the cognitive mechanisms underlying proximal context effects warrant more attention. Still, the general picture that currently emerges from this literature, in combination with the present findings, is that durational contrast would seem to play a limited role in both proximal and distal context effects.

This does not mean that general auditory accounts of speech perception, in which durational contrast plays a central role, should be discarded. Rather than conflicting with general auditory accounts of speech perception, this study supports these accounts by proposing another, neurobiologically plausible, general auditory mechanism to underlie rate-dependent perception. Neural entrainment to the amplitude modulations in the acoustic signal is introduced as explanatory mechanism behind the observed effects of presentation rate. Faster tone sequences are suggested to induce neural entrainment at a higher frequency, leading to a denser sampling regime, resulting in overestimation of segmental durations. However, empirical evidence of differential neural tracking inducing rate-dependent perception has not yet been provided. Neuroimaging experiments are currently being carried out to fill in this hiatus in our understanding of how speech rate shapes perception.
